# A new species of Rhodocybe sect. Rufobrunnea (Entolomataceae, Agaricales) from Italy

**DOI:** 10.3897/mycokeys.36.27094

**Published:** 2018-07-10

**Authors:** Alfredo Vizzini, Renato Jonny Ferrari, Enrico Ercole, Alessandro Fellin

**Affiliations:** 1 Department of Life Sciences and Systems Biology, University of Torino, Viale P.A. Mattioli 25, I-10125, Torino, Italy; 2 Santo Stefano 46, I-39030, San Lorenzo di Sebato (BZ), Italy; 3 Via G. Canestrini 10/B, I-38028, Revò (TN), Italy

**Keywords:** Agaricomycetes, Basidiomycota, Molecular markers, Phylogeny, Taxonomy

## Abstract

*Rhodocybe fumanellii* is described from Italy as a new species based both on morphological and molecular nrITS/nrLSU data. It belongs in sect. Rufobrunnea and is characterised by massive tricholomatoid basidiomata with reddish-brown tinges, adnate and crowded lamellae, an enlarged stipe base with long rhizomorphs, long sinuose slender cheilocystidia, ellipsoid basidiospores and the presence of caulocystidia. Drawings of the main micromorphological features as well as a colour photograph of fresh basidiomata *in situ* are provided and its morphological relationships with allied species are discussed.

## Introduction

Recently, Kluting et al. (2014), using a multigene phylogenetic analysis, redefined the classification of genera within the Entolomataceae. In particular, they proved that the genus *Rhodocybe* Maire, as morphologically delimited (Baroni 1981, Singer 1986, Noordeloos 1988, 2012) is heterogeneous, and it actually consisted of four lineages, of which *Rhodocybe* s.s., *Clitocella* Kluting, T.J. Baroni & Bergemann, *Clitopilopsis* Maire and *Rhodophana* Kühner should be considered as separate genera. *Rhodocybe* was restricted to the species possessing variable basidiomata (pleurotoid, collybioid, mycenoid, clitocyboid or tricholomatoid), variously coloured, white, grey, brown, pinkish, reddish, yellowish or combinations of these colours; lamellae variously attached, ranging from adnexed to adnate or (sub-)decurrent; basidiospores are thin-walled and evenly cyanophilic, angular in polar view with 6–12 facets and have pronounced undulate pustulate ornamentations in face and profile views. Hymenial cystidia are present or absent and, when present, they can be as pseudocystidia with brightly coloured contents or as hyaline leptocystidia found as cheilocystidia and sometimes as pleurocystidia and clamp connections are absent.

Within Rhodocybe s.s., section Rufobrunnea, typified by *R. roseiavellanea* (Murrill) Singer, is characterised by a reddish-beige, salmon pink, pinkish-brown or ochre pileus, adnate or decurrent lamellae and absence of pseudocystidia (Baroni 1981, Singer 1986, Noordeloos 2008, 2012). The section was later shown to be natural (monophyletic), also on a molecular basis (Kluting et al. 2014, Sesli and Vizzini 2017). The aim of the present paper is to describe a new species of Rhodocybe sect. Rufobrunnea from Italy circumscribed on both morphological and molecular data.

## Materials and methods

### Morphology

Macroscopic description was based from detailed field notes on fresh basidiomata. Colour terms in capital letters (e.g. Pompeian Red, Plate XIII) are those of Ridgway (1912). Fresh basidiomata were photographed *in situ* with a Nikon D5600 digital camera and then dried, while the photos of the microscopical structures, on which the line drawings were based, were obtained through a Zeiss Axiolab light microscope and an OPTIKAM B5 digital camera.

Micromorphologic features were observed on fresh and dried material; sections were rehydrated in distilled water or 3% NH_4_OH and then mounted in anionic Congo red as universal dye, lactic Cotton blue to test for cyanophily and Melzer’s reagent to determine amyloidity, separately.

All microscopic measurements were carried out with a 1000× oil immersion objective using the Optika Vision Lite 2.1 software. Basidiospores were measured from hymenophores of mature basidiomes and dimensions (hilar appendix excluded) are given as: (minimum–) average minus standard deviation – *average* – average plus standard deviation (–maximum) of length × (minimum–) average minus standard deviation – *average* – average plus standard deviation (–maximum) of width, Q = (minimum–) average minus standard deviation – *average* – average plus standard deviation (–maximum) of ratio length/width. Spore statistics were produced with R version 3.4.4 (R Core Team 2018). The following abbreviations are used: L = number of lamellae reaching the stipe, l = number of lamellulae between each pair of lamellae, Q = the basidiospore quotient (length/width ratio). Herbarium acronyms follow Thiers (2018).

### DNA extraction, PCR amplification, and DNA sequencing

**Figure 1.**
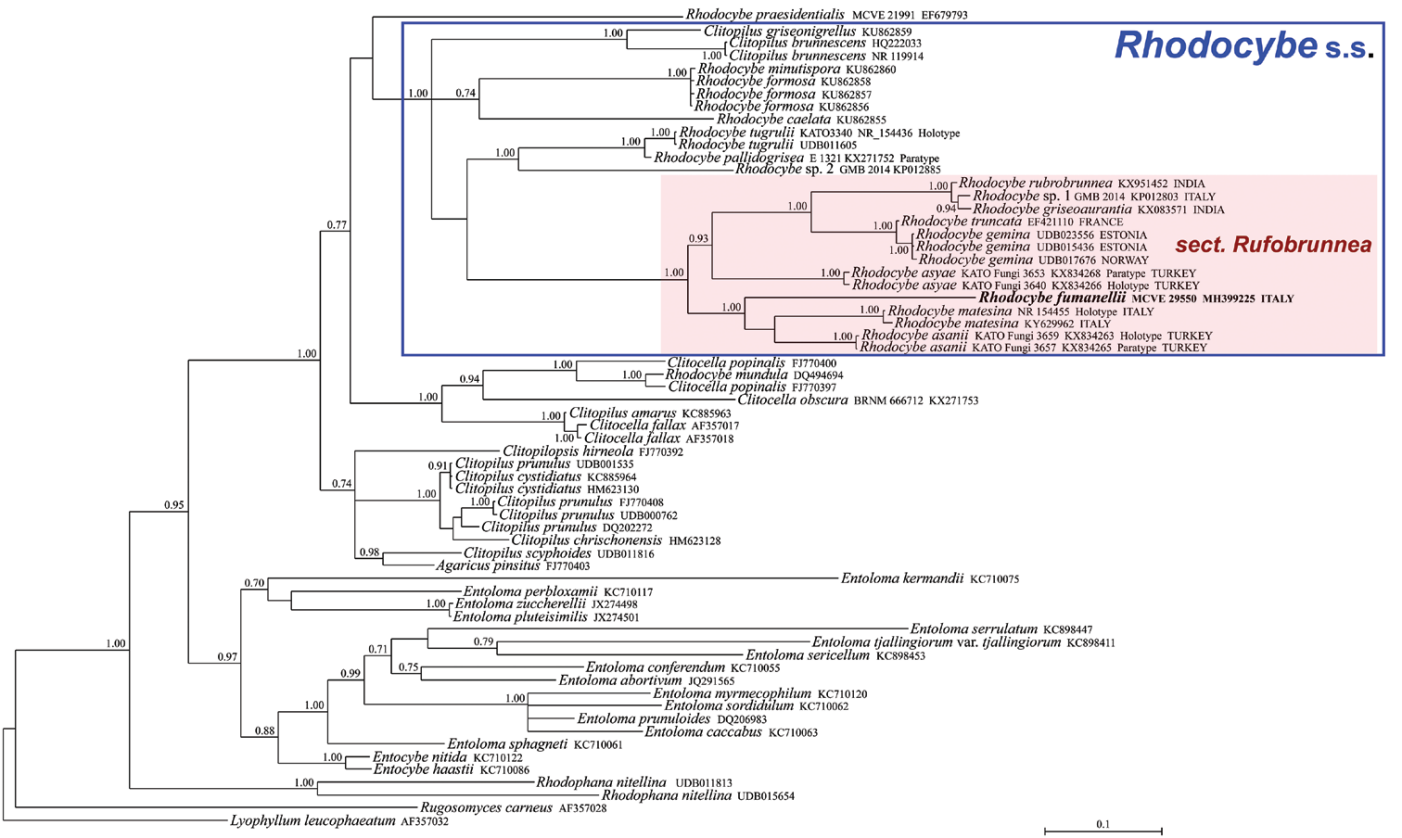
Bayesian phylogram based on the nrITS sequences of Entolomataceae, with Rugosomyces (Calocybe) carneus and *Lyophyllum leucophaeatum* as outgroup taxa. Only BPP values ≥ 0.70 are shown. The newly sequenced collection is in bold.

**Figure 2.**
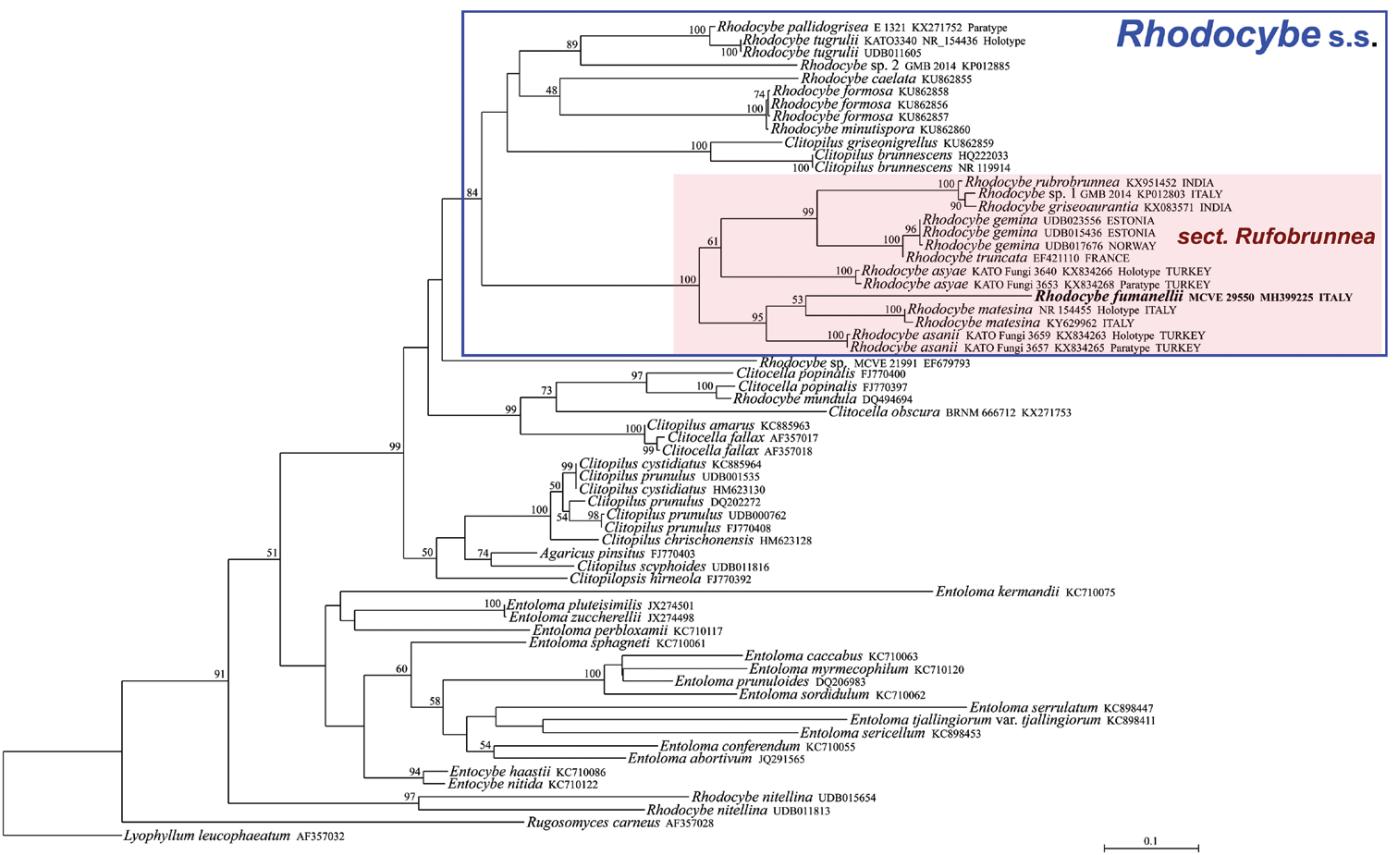
Maximum Likelihood phylogram based on the nrITS sequences of Entolomataceae, with Rugosomyces (Calocybe) carneus and *Lyophyllum leucophaeatum* as outgroup taxa. Only MLB values ≥ 50% are shown. The newly sequenced collection is in bold.

**Figure 3.**
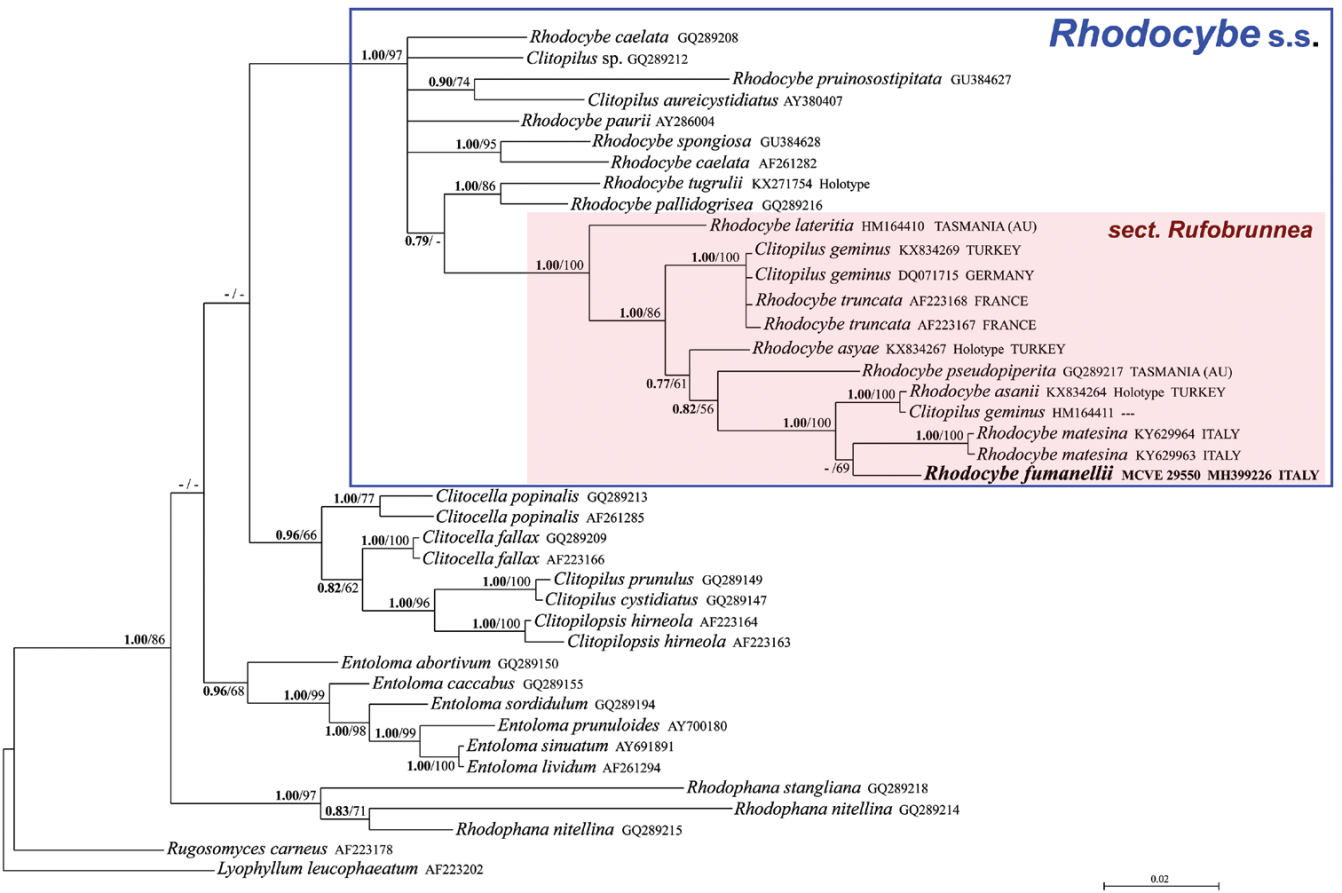
Bayesian phylogram based on the nrLSU sequences of Entolomataceae, with Rugosomyces (Calocybe) carneus and *Lyophyllum leucophaeatum* as outgroup taxa. Only BPP values ≥ 0.70 and MLB values ≥ 50% are shown. The newly sequenced collection is in bold.

Total DNA was extracted from a dry basidioma (MCVE 29550) by blending a portion of it (about 20 mg) with the aid of a micropestle in 600 μl CTAB buffer (CTAB 2%, NaCl 1.4 M, EDTA pH 8.0 20 mM, Tris-HCl pH 8.0 100 mM). The resulting mixture was incubated for 15 min at 65°C. A similar volume of chloroform:isoamyl alcohol (24:1) was added and carefully mixed with the samples until their emulsion. It was then centrifuged for 10 min at 13,000 g, and the DNA in the supernatant was precipitated with a volume of isopropanol. After a new centrifugation of 15 min at the same speed, the pellet was washed in cold ethanol 70%, centrifuged again for 2 min and dried. It was finally re-suspended in 200 μl ddH_2_O. PCR amplification was performed with the primers ITS1F and ITS4 (White et al. 1990, Gardes and Bruns 1993) for the nrITS region, while LR0R and LR5 (Vilgalys and Hester 1990) were used to amplify the nrLSU region (28S). PCR reactions were performed under a programme consisting of a hot start at 95 °C for 5 min, followed by 35 cycles at 94 °C, 54 °C and 72 °C (45, 30 and 45 s respectively) and a final 72 °C step for 10 min. PCR products were checked in 1% agarose gel and positive reactions were sequenced with primer ITS4. Chromatograms were checked by searching for putative reading errors and these were corrected. The PCR products were purified with the Wizard SV Gel and PCR Clean-UP System (Promega) following manufacturer’s instructions and sequenced by MACROGEN Inc. (Seoul, Republic of Korea). Sequences were checked and assembled using Geneious 5.3 (Drummond et al. 2010) and submitted to GenBank (http://www.ncbi.nlm.nih.gov/genbank/). Accession numbers are reported in Figs 1–3.

### Sequence alignment, dataset assembly and phylogenetic analysis

Sequences obtained in this study were compared to those available in the GenBank (http://www.ncbi.nlm.nih.gov/) and UNITE (http://unite.ut.ee/) databases by using the Blastn algorithm (Altschul et al. 1990). Based on the Blastn results, sequences were selected according to the outcomes of recent phylogenetic studies incorporating *Rhodocybe* s.l. taxa (Kluting et al. 2014, Crous et al. 2017, Sesli and Vizzini 2017). The nrITS and nrLSU datasets were analysed separately. The combined nrITS/nrLSU phylogeny was not performed as most *Rhodocybe* s.l. collections in GenBank are not provided with both molecular markers. Alignments were generated for each nrITS and nrLSU dataset using MAFFT (Katoh et al. 2002) with default conditions for gap openings and gap extension penalties. The two alignments were imported into MEGA 6.0 (Tamura et al. 2013) for manual adjustment. The best-fit substitution model for each single alignment was estimated by both the Akaike Information Criterion (AIC) and the Bayesian Information Criterion (BIC) with jModelTest 2 (Darriba et al. 2012). The GTR + G model was chosen for both the nrITS and nrLSU alignments. Two Lyophyllaceae, Rugosomyces (Calocybe) carneus (AF357028 and AF223178) and *Lyophyllum leucophaeatum* (AF357032 and AF223202) were used as outgroup taxa in both the nrITS and nrLSU analyses following Crous et al. (2017) and Sesli and Vizzini (2017). The nrITS dataset was partitioned into ITS1, 5.8S and ITS2 subsets.

Phylogenetic hypotheses were constructed with Bayesian inference (BI) and Maximum Likelihood (ML) criteria. The BI was performed with MrBayes 3.2.6 (Ronquist et al. 2012) with one cold and three incrementally heated simultaneous Monte Carlo Markov Chains (MCMC) run for 10 million generations, under the selected evolutionary model. Two simultaneous runs were performed independently. Trees were sampled every 1,000 generations, resulting in overall sampling of 10,001 trees per single run; the first 2,500 trees (25%) were discarded as burn-in. For the remaining trees of the two independent runs, a majority rule consensus tree showing all compatible partitions was computed to obtain estimates for Bayesian Posterior Probabilities (BPP).

ML estimation was performed through RAxML 7.3.2 (Stamatakis 2006) with 1,000 bootstrap replicates (Felsenstein 1985) using the GTRGAMMA algorithm to perform a tree inference and search for a good topology. Support values from bootstrapping runs (MLB) were mapped on the globally best tree using the “-f a” option of RAxML and “-x 12345” as a random seed to invoke the novel rapid bootstrapping algorithm. BI and ML analyses were run on the CIPRES Science Gateway web server (Miller et al. 2010). Only BPP and MLB values ≥ 0.70 and ≥ 50%, respectively, are reported in the resulting trees (Figs 1–3). Branch lengths were estimated as mean values over the sampled trees.

## Results

### Phylogenetic analyses

The nrITS data matrix comprised 64 sequences (1 from the newly sequenced collection, 54 from GenBank and 9 from UNITE). The nrLSU data matrix comprised 40 sequences (1 from the newly sequenced collection and 39 from GenBank). As the Bayesian and Maximum Likelihood analyses of the nrITS sequences are conflicting with each other regarding the precise position of our species, it was decided to show them both (Figs 1–2); on the contrary, BI and ML analyses of the nrLSU sequences produced comparable and congruent topologies and, consequently, only the BI phylogram, with both BPP and MLB values is shown (Fig. 3). Both in the nrITS and nrLSU analysis, our collection clusters in the genus Rhodocybe s.s. within the section Rufobrunnea (Figs 1–3). In particular, it forms a strongly supported clade together with *R. asanii* Seslı & Vizzini and *R. matesina* Picillo & Vizzini where it occupies an independent but uncertain position with regard to the other two species.

### Taxonomy

#### Rhodocybe fumanellii

Taxon classificationFungiAgaricalesEntolomataceae

Ferrari, Vizzini & Fellin sp. nov.

825646

Figs 4 , 5

##### Holotype.

Italy. Veneto, Venezia, Chioggia, Riserva Naturale Integrale Bosco Nordio, 45°7'19.563"N, 12°15'38.046"E, 4 m a.s.l., mixed broadleaved forest with *Fraxinus ornus* and *Quercus ilex*, on consolidated dunes, 10 November 2017, Renato Jonny Ferrari & Enrico Bizio (MCVE 29550).

##### Etymology.

dedicated to Ezio Fumanelli, Italian mycologist, naturalist and photographer.

**Figure 4.**
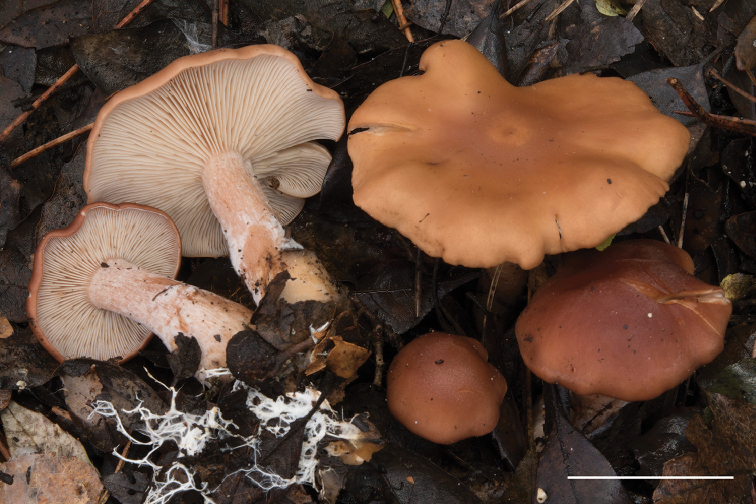
*Rhodocybe fumanellii* (MCVE 29550). Fresh basidiomes *in situ*. Scale bar: 50 mm. Picture by R.J. Ferrari.

*Habit* tricholomatoid (Fig. 4). *Pileus* 35–100 mm diam, at first convex with large central umbo, soon plane, irregular, with margin slightly inrolled when young, soon plane, strongly undulate, lobate when old, not striate, surface smooth, dry, greasy when wet, not or very slightly hygrophanous, at first reddish-brown (Nopal Red, Brazil Red, Plate I; Pompeian Red, Plate XIII; *Vinaceous-Rufous, Plate XIV) then brick-red (*Brick Red, Plate XIII), light orange to ochre (Flesh Ocher, Apricot Buff, Plate XIV) when old. *Lamellae* narrow, adnate, quite crowded (L = 60–80), intermixed with lamellulae of variable length [l = 1–3(–4)], up to 3–4 mm high, at first whitish-cream (Seashell Pink, Plate XIV; Pale Ochraceous-Salmon, Plate XV), finally pinkish (Pale Salmon Colour, Pale Flesh Colour, Plate XIV) when very old, with an irregular-eroded concolorous edge. *Stipe* 40–70 × 5–15 mm, cylindrical-clavate (at base to 20–28 mm broad), central, solid, pinkish (Light Corinthian Red, Plate XXVII; Light Congo Pink, *Vinaceous-Pink, Plate XXVIII), covered with a white flocculent-pruinosity, denser towards the apex, the base with a white dense mycelial tomentum and numerous thick white rhizomorphs. *Context* whitish, pink shaded, marbled, thicker (up to 9 mm) in the disc and thinner in the rest of the pileus, *odour* aromatic of walnut kernel, a little floury, *taste* mild, flour-aromatic, not astringent. *Spore-print* pinkish. *Macrochemical reactions* (on fresh material): 30% KOH on context and pileus surface negative.

**Figure 5.**
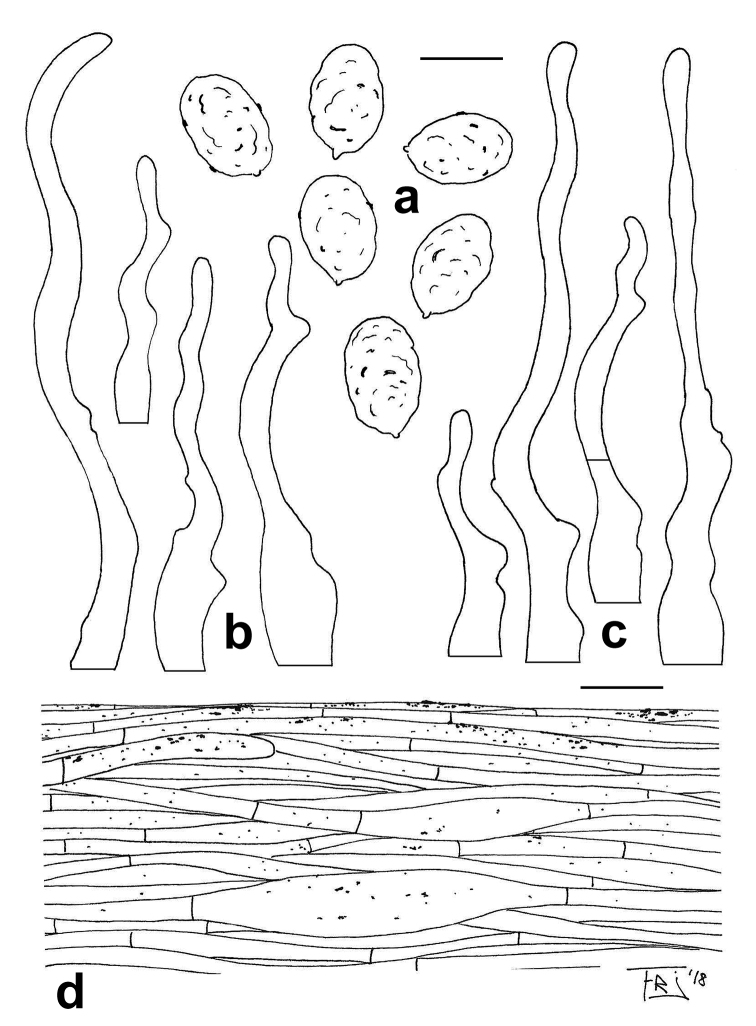
*Rhodocybe fumanellii* (MCVE 29550). Microscopical features. **a** Basidiospores **b** caulocystidia **c** cheilocystidia **d** pileipellis. Scale bars: 5 μm. Line drawings by R.J. Ferrari.

*Basidiospores* (5.3–)5.68–*6.26*–6.83(–7.3) × (3.5–)3.93–*4.26*–4.58(–5.1) µm (n = 40), Q = (1.22–)1.34–*1.47*–1.60(–1.78), ellipsoid, colourless under the light microscope, finely warty, pustulate, with a wavy profile (angular in polar view with 8–12 facets), walls cyanophilic, inamyloid (Fig. 5a). Lamella edge heterogeneous. *Basidia* 30–40 × 6.5–7 μm, clavate, 4-spored, thin-walled, sterigmata up to 5 μm long. *Basidioles* 30–45 × 4.5–6 μm, clavate. *Cheilocystidia* 35–95 × 3–6.5 μm, scattered, slender, flexuose-cylindrical, sometimes with protuberances and 1–2-septate, thin-walled (Fig. 5c). *Pleurocystidia* absent. *Hymenophoral trama* subregular, consisting of cylindrical parallel hyphae (2.5–5 μm) mixed with short, inflated, up to 13 μm wide elements. *Pileipellis* as a xerocutis, made up of subparallel, thin-walled hyphae, 2‒5 μm wide, orange-brown (in H_2_O), with presence of granular epiparietal pigment (observable in H_2_O and NH_4_OH), terminal elements obtuse (Fig. 5c). *Caulocystidia* (25–)30–50(–69) × (2.5–)3–4(–5) µm, slender with a cylindrical-irregular shape, thin-walled (Fig. 5b). *Clamp-connections* absent everywhere.

##### Habit, habitat and distribution.

In small groups (gregarious), in the litter of broadleaved trees on sandy soil. So far, known only from the type locality.

## Discussion

Section Rufobrunnea is a character-poor taxon with many species macroscopically very similar (cryptic species) and differing only in very subtle features (e.g. habit, basidioma colour, type of lamellae insertion, odour, taste, presence/absence of rhizomorphs) (Baroni 1981, Sesli and Vizzini 2017). To what extent these characters can be influenced by the environmental factors still remains to be verified. Also microscopic identification depends on observations of a rather limited set of characters, such as presence/absence of cheilocystidia, cystidial shape, spore size and ornamentation and pileipellis structure and pigmentation. From a traditional morphological perspective, this often makes species identification difficult or even daunting. Despite this, the species in this section are quite distinct if analysed in light of ribosomal sequences (Crous et al. 2017, Sesli and Vizzini 2017).

*Rhodocybe fumanellii* has proved to be an independent and distinct species within this section based on molecular analyses (Figs 1–3). Morphologically, it is circumscribed in having robust and massive basidiomata with a tricholomatoid habit and reddish-brown tinges, adnate not decurrent and crowded lamellae, an enlarged stipe base with evident long rhizomorphs, very long and slender cheilocystidia (up to 95 μm long), ellipsoid basidiospores (average Q = 1.47) and presence of caulocystidia.

The phylogenetically closest species to *R. fumanellii* are the recently described *R. matesina* and *R. asanii*. *Rhodocybe matesina* from Italy differs in a collybioid and slender habit (stipe up to 9 mm broad), thin context, a strongly hygrophanous pileus without reddish tinges, a smell similar to *Hygrophorus penarioides* Jacobsson & E. Larss., bitter and astringent taste, absence of rhizomorphs and an olive-green reaction on the pileus surface with KOH; microscopically *R. matesina* is distinguished due to shorter cheilocystidia (16.5–23 × 3–6.5 μm), the absence of caulocystidia and the presence in the pileipellis of rare pseudoclamps (Crous et al. 2017). *Rhodocybe asanii* from Turkey has a collybioid habit with a 20–45 mm broad pileus, thin and very fragile context (up to 4 mm thick at pileus centre), quite distant sinuate lamellae (L = 40–50), a stipe without rhizomorphs, indistinct odour and taste, smaller spores (5.8 × 4.1 μm on average, apiculus included), no cheilocystidia and caulocystidia and growth between debris and grass in coniferous woods (*Pinus* L., *Picea* A. Dietr., *Abies* Mill.) (Sesli and Vizzini 2017).

Hereafter, distinctive features of the species in the section Rufobrunnea that somehow morphologically resemble *R. fumanellii*, are provided. *Rhodocybe lateritia* T.J. Baroni & G.M. Gates described from Tasmania, is circumscribed by a burnt sienna or reddish-brown cup-shaped, up to 120 mm broad pileus, large, 5.5–11 × 4.5–7.5 μm basidiospores and ascending, cystidioid elements (pileocystidia) in the pileipellis (Baroni and Gates 2006, Noordeloos and Gates 2012). *Rhodocybe alutacea* Singer from North America has a smaller umbilicate hygrophanous pileus (up to 35 mm diam and up to 2 mm thick context) minutely erect-squamulose to subtomentose-squamulose at centre and thinner stipe (2.5–5 mm broad), a pileus margin remaining inrolled to incurved, decurrent lamellae, a farinaceous odour and taste (mild), larger spores (up to 8 × 5.5 μm) and shorter septate cheilocystidia (20–35 × 6.5–7 μm) with often capitulate terminal elements (Singer 1946, Baroni 1981, Baroni and Horak 1994). *Rhodocybe asyae* Seslı & Vizzini from Turkey differs in having smaller basidiomes (pileus up to 30 mm diam and stipe up to 5 mm diam), decurrent lamellae, stipe without rhizomorphs, mainly 2-spored basidia, no caulocystidia, less elongated basidiospores (average Q = 1.3) and grows in the litter of coniferous trees (*Pinus*, *Picea*, *Abies*) (Sesli and Vizzini 2017). *Rhodocybe incarnata* T.J. Baroni & Halling, from cloud forests in Venezuela, mainly differs by a pileus at first fire red, flame red, flame scarlet then becoming paler, matted subtomentose to matted pubescent, white to yellowish-white lamellae, shorter basidiospores (5.7 μm long on average) and cheilocystidia (14.6–25.9 × 2.4–4 μm) and pileipellis as a trichoderm (Baroni and Halling 1992). *Rhodocybe pseudopiperita* T.J. Baroni & G.M. Gates from Tasmania is distinguished by a weakly umbonate pileus with shallow depression around the umbo, indistinct odour or like mown grass, the presence of scattered cystidioid elements in the pileipellis and dimorphic basidiospore morphology with most of them being distinctly undulate-pustulate and smaller (5.5–6.5 × 4–5 μm) while ca. 30–45% of the basidiospores are almost smooth and distinctly larger (7–9 × 5–5.5 μm) (Baroni and Gates 2006, Noordeloos and Gates 2012). The North American *R. roseiavellanea* shares with *R. fumanellii* a robust habitus (not hygrophanous thick-fleshed pileus 35–70 mm broad and stipe 30–60 × 10–25 mm), a mild taste, a growth under oaks, but is distinguished by short decurrent to decurrent lamellae, a stipe without rhizomorphs, shorter cheilocystidia, 12–25 × 2–4 μm and large ellipsoid to subamygdaliform spores, (6.5–)7–9(–10) × (4–)5–5.5(–7) μm (Baroni 1981). *Rhodocybe gemina* (Paulet) Kuyper & Noordel. from Europe, Algeria, Morocco and Turkey differs considerably in having broadly adnate to subdecurrent lamellae, subglobose to broadly ellipsoid, 5–6.5(–7) × 4–5(–5.5) μm basidiospores and mainly growing in montane coniferous forests (above all *Picea* spp.) (Maire 1924, Malençon and Bertault 1975, Watling and Gregory 1977, Baroni 1981, Breitenbach and Kränzlin 1995, Noordeloos 1988, 2012, Sesli and Vizzini 2017). Rhodocybe gemina var. mauretanica (Maire) Bon and var. subvermicularis (Maire) Quadr. & Lunghini [= *Rhodocybe subvermicularis* (Maire) Ballero & Contu] from European Mediterranean areas, Algeria and Morocco show a collybioid to clitocyboid habit with a pileus up to 50 mm broad and decurrent lamellae and no evident cheilo- and caulocystidia (Maire 1924, Malençon and Bertault 1975, Baroni 1981, Bon 1990, Ballero et al. 1992). Finally, *R. nuciolens* (Murrill) Singer from North America shows slender basidiomes with short decurrent subdistant lamellae, a 2–9 mm broad stipe, context up to 3 mm thick at pileus centre, large ellipsoid to amygdaliform basidiospores 5.5–8(–9) × (3–)4–5(–5.5) μm, and it grows in humus, sandy soil or on decaying wood under *Pseudotsuga menziesii*, *Sequoia sempervirens*, *Abies* sp., or *Arbutus menziesii* (Murrill 1913, Singer 1946, Baroni 1981).

## Supplementary Material

XML Treatment for Rhodocybe fumanellii
